# Immunodominant antiviral T cell responses outcompete immuno-subdominant antitumor responses to reduce the efficacy of oncolytic viroimmunotherapy

**DOI:** 10.21203/rs.3.rs-6131273/v1

**Published:** 2025-03-18

**Authors:** Richard Vile, Benjamin Kendall, Olivia Liseth, Thanich Sangsuwannukul, Natalie Elliott, Maria Chiriboga Yerovi, Jill Thompson, Jack Swanson, Soha Rizk, Rosa Diaz, Jason Tonne

**Affiliations:** Mayo Clinic; Mayo Clinic; Mayo Clinic; Mayo Clinic; Mayo Clinic; Mayo Clinic; Mayo Clinic; University of Minnesota; Mayo Clinic; Mayo Clinic; Mayo Clinic

**Keywords:** CD8 T cells, Oncolytic virotherapy, Vesicular Stomatitis Virus, Immune checkpoint blockade, antiviral immunity, antitumor immunity, immunotherapy

## Abstract

The paradigm in the field of oncolytic virotherapy proposes that tumor cell killing by an oncolytic virus (OV) culminates in the priming of antitumor CD8 T cells. However, this ignores the impact a highly immunodominant antiviral response against the OV has on the antitumor response which has been weakened by mechanisms of central tolerance. Here, we show that inflammatory Vesicular Stomatitis Virus (VSV) failed to prime an adoptively transferred, or pre-existing, population of tumor-reactive T cells. Combination with αPD1 immune checkpoint blockade therapy improved survival only when VSV expressed tumor associated antigens (TAA). These data show that, in this model, the highly inflammatory OV VSV alone actively outcompetes antitumor immunity. However, we also show that viral expression of a mutant near-self TAA can break central tolerance expanding heteroclitic self-reactive and near-self-reactive T cells, thus overcoming viral immunodominance by promoting tumor-specific T cell proliferation in parallel with expanding antiviral T cells.

## Introduction

There is a sizeable gap in the field of viral immunotherapy in the understanding of the relative dynamics, and interplay between, antiviral and antitumoral CD8 T cell expansion during oncolytic virotherapy. Oncolytic viruses (OVs) are immuno-oncology agents engineered specifically to replicate in and kill tumor cells. The current paradigm proposes that OV tumor cell killing induces immunologic cell death which triggers innate responses through exogenous genome-sensing pathways such as Toll like receptors (TLRs) and the STING/cGAS pathway, culminating in the priming and expansion of an adaptive antitumor T cell response^1–4^. A particularly appealing feature of this model is that the inflammatory killing of tumor cells with the release of tumor associated antigens (TAAs) could synergize with cytotoxic T-cell-focused strategies such as immune checkpoint blockade (ICB) and CAR T cell therapy for the treatment of solid tumors, (reviewed in ^5–8^). However, complete tumor clearance by OV monotherapy remains challenging due to its requirement for complete infection of the tumor in the face of multiple tumor- and stroma-associated anti-viral mechanisms, coupled with its reliance on a T cell bystander effect to clear uninfected cells. These barriers to successful OV monotherapy have accelerated efforts to understand how best to induce tumor specific T cells for a durable antitumor memory response since insufficient priming and expansion of tumor specific T cells following OV therapy may be a contributing factor to reduced survival outcomes^9^.

Poorly immune cell-infiltrated (immunologically “cold”) solid tumors evade the immune response using a variety of mechanisms. These include downregulation of major histocompatibility complexes (MHC) preventing tumor antigen presentation, or recruitment of anti-inflammatory tumor associated macrophages and regulatory T cells, which collectively hinders CD8 T cell targeting and fitness^10,11^. Inflammatory OVs effectively reverse the immunosuppressive tumor microenvironment (TME) and dramatically increase the infiltration of CD45+ immune cells. However, this increase in CD45+ T cell infiltration does not necessarily reflect the *de novo* priming of tumor-specific T cells. In contrast, it most likely reflects the priming and recruitment of an antiviral T cell response. This viral immunodominance is determined by antigen affinity and concentration, MHC availability, and dwell time at the immune synapse – collectively referred to as antigen avidity^12,13^. It follows that a high concentration of high affinity non-self viral antigens is much more likely to produce cytotoxic lymphocytes (CTL) targeting viral proteins as opposed to the inherently lower-affinity tumor self-antigens. As such, the goal of inducing a polyvalent CTL response to both viral and tumor antigens following OV therapy is complex and requires multiple, often competing, interactions at the level of immune priming and T cell expansion, as evidenced by the barriers faced in cancer vaccine development even in the absence of competing viral responses^14–16^. Successful therapy often requires the breaking of central tolerance to self-antigens as it is required for CTL-mediated TAA-specific immunity^17–19^. To address this, we, and others, have shown the importance of directly expressing TAAs from the viral vector to boost the antitumor response, whether by increasing the concentration^20,21^ and variety of TAAs or directly manipulating them to increase immunogenicity^20,22,23^. These strategies provide promising solutions for generating tumor-specific immunity which can be further boosted by ICB^24,25^. Nonetheless, the underlying distracting effect of the antiviral response upon the generation of an effective anti-tumor response remains a major impediment to the efficacy of OV therapy as immunotherapy.

We have previously reported that OV monotherapy using highly inflammatory Vesicular Stomatitis Virus (VSV) can lead to the attrition of tumor specific cytotoxic T cells resulting in a lack of synergy with αPD1 ICB therapy against B16 melanoma^26,27^. In an ICB-sensitive tumor model of hepatocellular carcinoma (HCC) we also observed a significant reduction of antitumor therapy when OV and aPD-L1 ICB were combined compared to ICB alone^25^. These findings contrast with studies which observed antitumor T cell expansion using DNA viral vectors such as Adenovirus DNX-2401^28,29^ and Vaccinia JX-594^30^, and relatively slower-replication RNA vectors such Newcastle Disease Virus (NDV)^31,32^, in several tumor models^28,29,31–34^. Understanding this disparity will come from a detailed understanding of the immunological consequences of oncolytic virus treatment with respect to the interaction between the potent antiviral and antitumoral host response.

Therefore, here we directly addressed the impact of oncolytic virotherapy on the host antitumor T cell response. We hypothesized that an immune response stimulated by a highly immunogenic oncolytic virus immunologically distracts from the priming, proliferation, and cytotoxic activity of antitumor CD8 T cells and consequently restricts therapeutic efficacy. We show in the syngeneic B16 melanoma and MC38 colon adenocarcinoma models that the highly inflammatory VSV was unable to stimulate an antitumor T cell response in immunocompetent C57 mice even under largely optimal immunological conditions where the TAA was a non-tolerized, highly immunogenic antigen. However, when the virus was itself armed with the tumor associated antigen (VSV-TAA) a potent antitumor T cell response was generated. Moreover, treatment with the control virus led to the ablation of active antitumor T cells, and a shift of the tumor-reactive T cell landscape toward exhaustion. This shift in phenotype correlated with decreased survival, and combination with αPD1 ICB therapy significantly improved mouse survival only in mice treated with VSV-TAA compared to VSV wildtype (VSV-wt). Treatment with single-cycle Adenovirus serotype 5 had similar effects, with anti-tumor T cells only expanded when the vector encoded the TAA. However, by expressing mutated, altered-self versions of a TAA we identified a goldilocks affinity threshold of TAA recognition by T cells which generated T cells which cross reacted with unaltered, self TAA expressed by the tumor. Presence of these heteroclitic T cells correlated with improved tumor control and the phenotype of these T cells showed distinct profiles of tumor experience. Taken together, our data challenges the paradigm that immunogenic viruses alone function as *in situ* tumor vaccines. However, we offer a potential strategy to enhance anti-tumor T cell responses which retains the acute antitumor therapy driven by antiviral innate and adaptive responses, whilst alleviating the antiviral T cell distraction that diminishes the induction and efficacy of curative tumor-specific T cells.

## Results

### Oncolytic VSV is unable to expand high-affinity antitumor OT-1 T cells *in vivo*.

**Figure 1:**
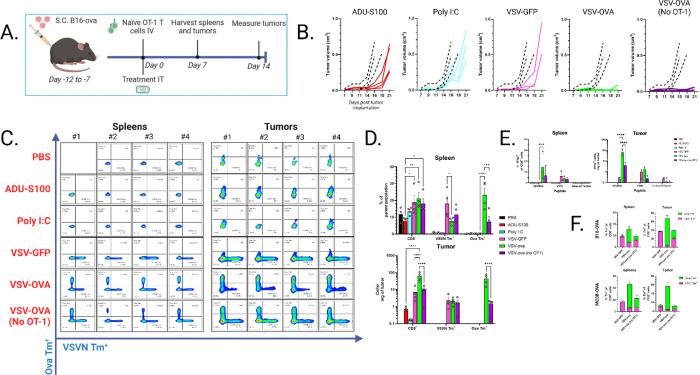
A) Schematic of *in vivo* study. C57 B6.SJL-Ptprca Pepcb/BoyJ (“Pepboy”) mice (CD45.1+) were implanted subcutaneously (s.c.) with 5e5 B16-OVA cells. 1e6 CD8α cells enriched from congenic OT-1 (CD45.2+) splenocytes were administered IV. Antiviral immune adjuvants (Poly:IC (50ug) and ADU-S100 (100ug)) or 5e8 pfu VSV were administered intratumorally (i.t.). B) Tumor growth kinetics versus PBS control (black dotted line) (n=4). C-D) Splenocytes and tumors were harvested at 7dpi and fluorescent tetramer-based flow cytometry analysis was performed to determine T cell specificity. E) Splenocytes and tumor cells were restimulated ex vivo with Ova_257–264_ (SIINFEKL) or VSVN_52–59_ (RGYVYQGL) peptides and detection of IFNγ expression was determined via intracellular staining. Reo μ1_133–140_ (VSPKYSDL) peptide was used as an irrelevant peptide control. F) Experiment (A) was repeated in the MC38-ova tumor model, and tetramer staining for T cell specificity was performed as before. n=4. Error bars indicate standard error of the means (SEM). P values were determined using a two-way ANOVA with a Tukey multiple-comparison posttest. Statistical significance was set with * indicating a *p* value less than 0.05, ** <0.01, *** <0.001, and **** <0.0001.

To explore the *de novo* priming of antitumor T cells following OV therapy with VSV, we adoptively transferred naïve OT-1 T cells into mice bearing the immunologically cold B16 melanoma tumor model expressing the artificial tumor antigen ovalbumin (B16-OVA). We employed fluorescent H2-K^b^ MHCI tetramers bound to either a VSV nucleoprotein (VSVN)-derived epitope or the SIINFEKL epitope of ovalbumin (OVA) to precisely identify expansion of 1) the adoptively transferred OT-1 T cells; 2) endogenous, OVA-reactive T cells and 3) antiviral T cells. Immunocompetent mice bearing B16-OVA melanoma tumors were treated with OV or non-viral immune adjuvants to characterize the possible competing role of viral proteins in the expansion of tumor reactive T cells (Fig. 1A–B). An immunogenic dose of innate immune adjuvants, which have previously shown to increase immune infiltration without ablating the tumor ADU-S100^35^ or Poly I:C^36^, did not generate a detectable OVA-specific T cell population as determined by SIINFEKL tetramer staining (Fig. 1C–D).These data suggest that triggering of the TLR3 or the STING/cGAS pathways does not induce sufficient tumor cell lysis to prime an OT-1 immune response even though in this system the OVA antigen is a fully foreign, non-tolerized TAA. A significant population of SIINFEKL-reactive T cells was detected when VSV expressed TAA (VSV-OVA). However, in contrast, no antitumor T cells could be detected following treatment with VSV alone (VSV-GFP) despite the induction of a potent anti VSVN T cell response (Fig. 1C–D). T cell functionality was confirmed via stimulation of lymphocytes isolated from spleens and tumors with SIINFEKL peptide or VSVN peptide epitope RGYVYQGL and interferon gamma (IFNy) was detected, mirroring the conclusions from the tetramer data (Fig. 1E). We then repeated this study in a subcutaneous MC38 colon adenocarcinoma tumor model and found similar results, confirming our findings in both immunologically cold (B16) and warm (MC38)^37^ subcutaneous tumors (Fig. 1F). Of note, we observed equivalent expansion of antiviral (VSVN) and endogenous antitumor T cells, highlighting the parallel expansion of T cells against equally foreign VSVN and OVA generated by the VSV-OVA vector. These data show that VSV therapy was unable to expand either a pre-existing or an endogenous high affinity antitumor T cell population in the context of an immunogenic, non-tolerized and non-self tumor antigen, suggesting a potentially inhibitory role of the antiviral response to the establishment of either endogenous or OT-1 antitumor T cells.

### Expansion of endogenous anti-OVA T cells is limited by an immunodominant OT-1 population.

**Figure 2:**
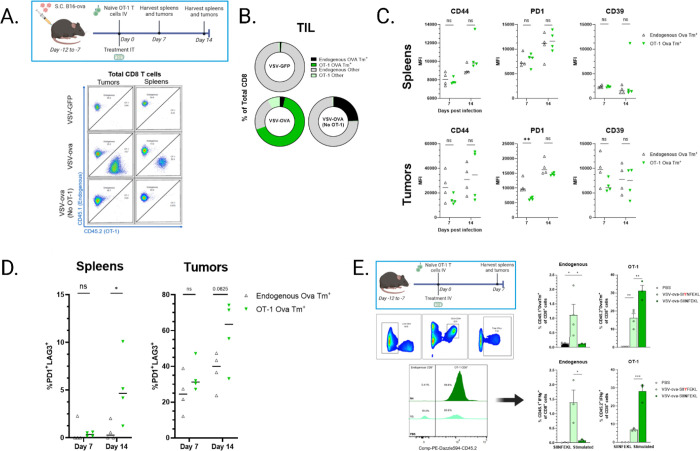
A) Schematic of *in vivo* study. Pepboy mice bearing s.c. B16-OVA tumors were treated with a single i.t. administration of 5e8 pfu VSV or PBS control, and tissues were harvested for flow cytometry analysis. Adoptively transferred OT-1 cells were enumerated via detection of CD45.2. n=4. B) T cell specificity at day 7 was determined via tetramer staining and mean values are plotted. Flow cytometry analysis for markers of CD8 T cell C) functional status and D) exhaustion from tissues harvested at days 7 and 14 post infection. n=4. E) Tumor-free Pepboy mice were treated i.v. with PBS or 1e7 pfu VSV-OVA (VSV-OVA-SIINFEKL), or 1e7 pfu VSV expressing ovalbumin mutation at the SIINFEKL epitope at position 3 (Y3). Endogenous and adoptively transferred cells were enumerated as before and SIINFEKL tetramer specificity was determined as before n=4. Splenocytes were stimulated *ex vivo* with SIINFEKL or SIYNFEKL peptide for detection of IFNγ expression. Unstimulated control values were subtracted from respective experimental group to control for constitutively active T cells. n=3. Error bars indicate standard error of the means (SEM). P values were determined using a one-way ANOVA with a Tukey multiple-comparison posttest. Statistical significance was set with * indicating a *p* value less than 0.05, ** <0.01, *** <0.001, and **** <0.0001.

Given the rapid expansion of both VSVN and OVA-specific T cells in response to VSV-OVA, we studied how this environment affects the endogenous anti-OVA response during VSV-OVA therapy. We stratified and quantified donor OT-1 T cells (congenic CD45.2^+^) and host T cells (B6.SJL-Ptprca Pepcb/BoyJ, CD45.1^+^) from mice treated with VSV-OVA (Fig. 2A). Interestingly, the absence of OT-1 T cells allowed for a greater expansion of endogenous anti-OVA T cells, indicating that adoptively transferred OT-1 T cells achieved dominance over the host anti-OVA T cell response (Fig. 2B). Splenocytes 7- and 14-days post VSV-OVA administration showed no significant difference of expression between OVA-specific subtypes in activation markers CD44 and Programmed Cell Death Protein 1 (PD-1), and we observed similar expression levels of CD39, an ATP hydrolase often associated with T cell experience in the tumor microenvironment (TME)^38^ (Fig. 2C). There was, however, a significantly lower median fluorescence intensity (MFI) of PD-1 in tumor infiltrating lymphocytes (TIL) at Day 7 indicating reduced expression of PD-1 per T cell, suggestive of improved T cell fitness in the TME. Dual expression of PD1 and lymphocyte activation gene 3 (LAG3) signifies functional exhaustion of T cells, and we noted a greater level of exhaustion in OT-1 T cells from splenocytes at 14dpi, although the disparity did not reach significance in the tumor (Fig. 2D). These findings suggest that adoptive transfer of OT-1 T cells inhibits the expansion of endogenous anti-OVA T cells and promotes their relatively earlier terminal exhaustion. but the competitive impact of adoptively transferred T cells on factors such as cytokine availability affecting the functionality of endogenous T cells requires further study. To determine if it was possible to alleviate the pressure of OT-1 dominance, we generated a VSV expressing a low affinity mutant form of OVA which has a single amino acid substitution in the 3^rd^ position of the SIINFEKL epitope (Fig. 2E). This peptide has a 4-fold lower functional affinity to an OT-1 TCR, and by extension, an endogenous SIINFEKL-specific TCR,^39^ which reduces the immediate expansion potential of the OT-1 T cells. Administration of OT-1 T cells and our VSV-Y3 variant led to a boosted expansion of endogenous SIINFEKL-reactive T cells, confirming that it is possible to exploit a competitive dynamic between coexisting groups of expanding T cells. These data indicate that the presence of a large competing T cell population places a ceiling of clonal expansion of a subdominant T cell population, although the precise mechanisms of this suppression remain unclear.

### VSV induces a shift in the T cell landscape distinct from tumor infiltrates stimulated by nonviral adjuvant.

**Figure 3:**
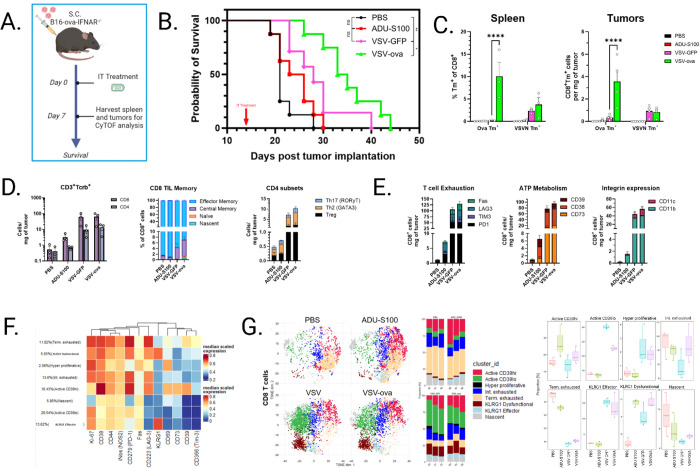
A) Schematic of survival and immunophenotyping study. C57Bl/6J mice implanted s.c. with 5e5 B16-OVA-IFNAR^−/−^ cells, then treated i.t. with STING adjuvant (ADU-S100) or OV, n=8. Statistical significance was determined via Gehan-Breslow-Wilcoxon test. C) Spleens and tumors were harvested at 7dpi and stained with fluorescent tetramers for flow cytometry analysis, n=4. P values were determined using a two-way ANOVA with a Tukey multiple-comparison posttest. Statistical significance was set with * indicating a *p* value less than 0.05, ** <0.01, *** <0.001, and **** <0.0001. D-E) Cytometry by time-of-flight analysis of tumor infiltrating lymphocytes and individual phenotype and lineage markers were assessed. n=3. Error bars indicate standard error of the means (SEM). F) Workflow of dimensionality reduction of tumor infiltrating CD8 T cells based on phenotype marker expression and subsequent generation of clusters. Annotation based on median expression of markers of proliferation, activation, and exhaustion as depicted via heatmap. G) t-Distributed Stochastic Neighbor Embedding (tSNE) plots based on expression of stratified by experimental condition and relative abundance of phenotype groups were plotted.

To better model the natural host immune response to OV in tumor bearing mice we repeated the previous study with immune adjuvant (ADU-S100) or VSV +/− OVA in the absence of OT-1 T cells and performed survival analysis (Fig. 3A–B). We observed marginally improved survival with STING agonist but treatment with VSV-GFP reached significance and the greatest survival benefit was observed in mice treated with VSV-OVA. Tetramer staining confirmed the expansion of antitumor T cells in the VSV-OVA group as before (Fig. 3C). Mass cytometry by time-of-flight (CyTOF) was employed on tumor infiltrating lymphocytes and individual marker expression was first assessed on CD3 T cells. We observed a striking ten-fold increase in CD8 T cells upon viral infection relative to STING agonist treatment, indicating the limitations of a single, low-dose immune adjuvant (Fig. 3D). VSV and VSV-OVA treatments comparably induced the expansion and/or infiltration of central memory (CD44^+^CD62L^+^) and conventional CD4 T cells along with CD25^+^FoxP3^+^ regulatory T cells and both viral treatments yielded comparable numbers of exhausted T cells, T cells expressing modulators of the TME, and integrin-expressing T cells often associated with an antiviral response (Fig.3E)^40,41^. Based on individual marker expression, it would appear that there is little phenotypic distinction amongst T cells expanded in the presence VSV-GFP and VSV-OVA. However, multidimensional clustering analysis illuminated unique CD8 T cell populations generated when VSV expresses TAA (Fig. 3F). Along with terminally exhausted T cells (PD1^hi^LAG3^hi^TIM3^hi^) we observed multiple populations of activated T cells, including TIM3^low^ intermediately exhausted T cells and those with differential expression of CD39 and killer cell lectin-like receptor G1 (KLRG1), a marker of a short-lived/dysfunctional effector T cell populations (Fig 3G). Cluster abundance analysis showed a greater expression of CD39^hi^ cells in ADU-S100 and VSV-OVA treated tumors compared to VSV-GFP treated tumors, suggesting that this is a functional population present in a tumor independent of viral infection. Although depleted by VSV, this population was partially rescued if the virus expressed TAA. We observed a population of dysfunctional KLRG1^hi^ T cells with reduced abundance following VSV-GFP treatment. Counterintuitively, lower abundance of CD39^hi^ and KLRG1 dysfunctional T cells with VSV-GFP treatment would imply an improved protective CD8 T cell repertoire. However, the lack of survival benefit outcome would suggest that these T cells lack the antigen specificity needed to clear tumor cells which were not infected. These data show the dramatic phenotypic shift of tumor infiltrating T cells towards an antiviral state at the expense of pre-existing, potentially tumor reactive T cell populations.

### Antiviral heat induced by different OVs modulates the antitumor T cell response but does not necessarily promote therapy.

**Figure 4:**
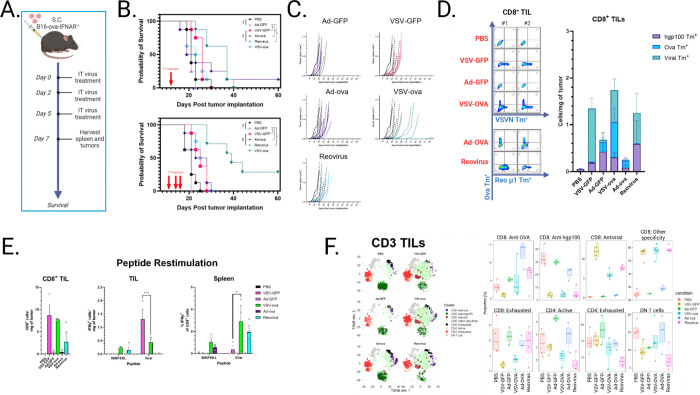
A) Schematic of in vivo study. B-C) Survival curves and tumor growth kinetics of C57BL/6 mice bearing s.c. B16-OVA-IFNAR^−/−^ tumors treated IT with 5e8 pfu VSV, 5e8 ifu Adenovirus, and 4.5e8 pfu Reovirus 1X or 3X. Statistical significance was determined via Gehan-Breslow-Wilcoxon test. D) Tissues from mice treated 3X i.t. were harvested 7 days post initial infection and stained with tetramers for tumor epitopes Ova_257–264_ (SIINFEKL) and hgp100_25–33_(KVPRNQDWL), and viral epitopes VSVN_52–59_ (RGYVYQGL) *or* Reo μ1_133–140_ (VSPKYSDL). Reo μ1 tetramer and peptide were used in the Ad-OVA treatment condition to serve as a negative control. n=3. E) Tumor cells and splenocytes were pulsed with SIINFEKL or viral peptides and IFNγ secretion was detected via intracellular staining. Unstimulated control values were subtracted from respective experimental group to control for constitutively active T cells. n=3. Error bars indicate standard error of the means (SEM). P values were determined using a one-way ANOVA with a Tukey multiple-comparison posttest. Statistical significance was set with * indicating a *p* value less than 0.05, ** <0.01, *** <0.001, and **** <0.0001. F) tSNE plots stratified by experimental condition and subclusters frequencies were plotted.

To test the connectivity of inflammation and CD8 T cell infiltration across other oncolytic virotherapies, we treated tumor bearing mice with Reovirus or replication-defective Adenovirus, both of which have been extensively characterized in preclinical and clinical settings^28,29,42–44^. As VSV is sensitive to type I interferons and B16 tumors have partially-intact IFN machinery^45^, we generated a B16-OVA interferon alpha receptor knockout cell line (B16-OVA-IFNAR^−/−^) to ensure maximal viral replication. Two separate experiments were performed using either single-dose or triple-dose intratumoral (IT) therapy to identify therapeutic impact based on viral load. A marginal improvement on survival using Ad5-OVA and Reovirus was observed, but neither were able to improve survival and restrict tumor growth compared to VSV-OVA (Fig 4A–C). Antigen specificity was determined using tetramers for VSVN, SIINFEKL, Reovirus u1, and melanoma antigen hgp100, which is the human variant of endogenous H2-D^b^-restricted melanoma antigen gp100. (Fig. 4D). Of note, although OVA-specific T cells were generated with Ad-OVA therapy, the overall poor infiltration of immune cells resulting from inherently limited adenoviral replication in murine cells possibly contributed to the poor survival outcome. Restimulation of splenocytes and TILs with TAA or viral peptide confirmed functional reactivity of clonally expanded T cells (Fig. 4E). Phenotypic analysis of CD3 T cells identified a large cluster of activated T cells in VSV and Reovirus-treated tumors, which are presumably antiviral in nature but not specific to the immunodominant Reo and VSVN peptides (Fig. 4F). T cells against low-affinity hgp100, relative to non-self OVA, also appeared to be present at baseline and in adenovirus groups but were depleted in inflammatory VSV and Reovirus mice, indicating a passive non-proliferative role of these infiltrating populations despite their tumor antigen specificity. Together, these data further emphasize the challenges faced by inflammatory OVs in expanding endogenous antitumor T cells in the absence of a virally-encoded high affinity tumor antigen.

### Combination VSV and αPD1 ICB eliminates tumor only when high affinity TAA is expressed by virus.

**Figure 5:**
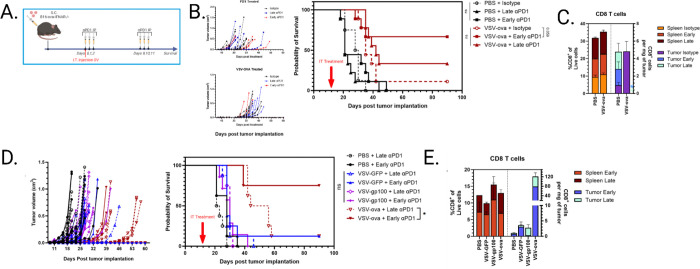
A) Schematic of OV + ICB treatment schedule. 3 treatments of 100ug αPD1 immune checkpoint blockade antibody or isotype control was administered IP at days 0–2 or 9–11 post infection. Tumors were injected i.t. with 5e8 pfu VSV at study day 0. n=9. B). Tumor growth of PBS or VSV-ova treated groups with corresponding survival plot. C) Enumeration of splenic and tumor infiltrating CD8+ T cells from (B) at 16dpi, n=2–3 mice per group. * = no tumors. D) Repeat of survival study (A) including VSV-GFP and VSV-hgp100 treatment conditions. n=8. Survival analyses utilized the Gehan-Breslow-Wilcoxon test to determine significance. E) Enumeration of splenic and tumor infiltrating CD8+ T cells from (D) at 16dpi, high mortality limited sample size. n=1–4.

Based on the paradigm that OV therapy generates potent antitumor T cell responses, VSV’s ability to increase CD8 T cell infiltration of the cold B16 melanoma tumor suggested that it may be an ideal candidate to be combined with immune checkpoint blockade (ICB) therapy. In light of our previous findings that VSV alone did not generate antitumor T cells, we hypothesized that delaying αPD1 ICB therapy until after the onset of antiviral T cells contraction, generally considered to be around day 7 post-infection, would better promote the survival of antitumor T cells expanded from viral or T cell-mediated lysis (Fig. 5A). The B16 melanoma model is refractory to ICB monotherapy as evidenced by the PBS+ICB control group (Fig. 5B). However, VSV-OVA therapy was greatly amplified by ICB depending on the timing of ICB administration (Fig. 5B–C). 67% of mice survived when αPD1 was administered early as opposed to 30% in late administration, possibly marking the contraction and/or exhaustion of tumor infiltrating T cells by day 9. To explore the impact of ICB timing in the absence of an immunogenic TAA, we then included VSV alone (VSV-GFP) to generate low levels of tumor antigen as a result of oncolysis – as well as VSV expressing hgp100 (VSV-hgp100) - which models the expression of a lower affinity near-self antigen compared to the highly artificial OVA. Consistent with our previous observations that the antiviral response can overwhelm endogenous, low affinity T cell responses, we saw no significant therapeutic benefit between VSV-GFP and VSV-hgp100 regardless of the timing of ICB administration (Fig. 5D), despite a more durable systemic CD8 T cell response being generated when treatment was administered early (Fig. 5E). Although we explored timing of ICB administration, we were unable to improve mouse survival using the highly inflammatory OV encoding low affinity tumor antigens, indicating that antigen affinity could play a crucial role in the face of the dominant antiviral response we have seen thus far.

### Virally-encoded near-self, heteroclitic tumor antigens resolve the conflict between dominant antiviral and sub-dominant anti-tumor T cell responses.

Our findings support the hypothesis that the immunodominant antiviral response can occur at the expense of an antitumor T cell population (Figs. 1–3) and that overcoming this dominance was only successful using a high-affinity, non-self tumor antigen expressed by OV (Fig. 5D–E). Therefore, we hypothesized that it would be possible to employ a heteroclitic TAA, whose affinity for naïve T cells would exceed the threshold required to break central tolerance to the endogenously expressed tumor antigen and enhance the immunogenicity of a self TAA in the context of OV delivery. Zehn et al. previously established an elegant system for study of TCR:antigen affinity where amino acid substitutions in the immunogenic epitope of OVA (SIINFEKL) incrementally reduce functional affinity to the cognate TCR without compromising its binding to MHC^39^. We implemented this model by expressing whole OVA protein bearing altered peptide ligands (APL) by our oncolytic VSV and validated its functional properties in C57 mice implanted with OT-1 T cells (Sup. Fig. 1). We subsequently treated RIP-mOVA mice – in which the OVA antigen is now a fully tolerized, self-antigen expressed predominantly on pancreatic beta cells – to attempt to break central tolerance to the tumor antigen.

**Figure 6:**
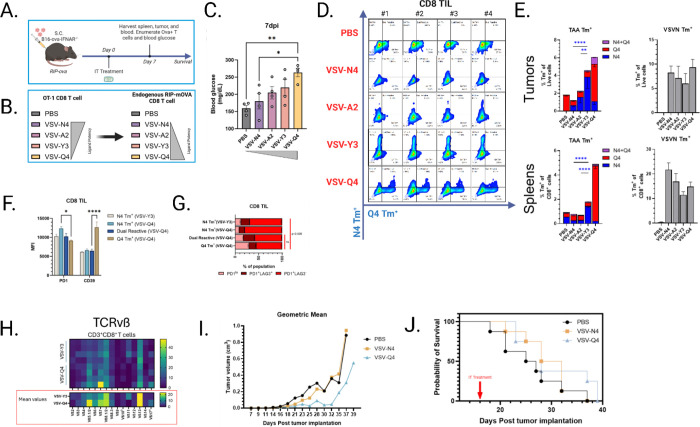
A) Schematic of VSV-altered peptide ligand (APL) *in vivo* study. RIP-mOVA mice implanted s.c. with 5e5 B16-OVA-IFNAR_−/−_ cells were treated i.t. 5e8 pfu VSV-APL. B) Projected ligand potency as determined by peptide reactivity of OT1 T cells and endogenous T cells in the RIP-mOVA model. C) Treated mice were fasted for 4h and blood glucose was measured. P values were determined using a one-way ANOVA with a Tukey multiple-comparison posttest. D-E) Tissues were harvested 7 days post virus administration and cells were stained with SIINFEKL and SIIQFEKL tetramer to determine T cell specificity. P values were determined using a two-way ANOVA with a Tukey multiple-comparison posttest, and significance relative to N4 Tm^+^ T cells was plotted. F-G) Activation phenotype of SIINFEKL T cells from Y3 treated mice and Tm+ T cells from Q4 treated mice was assessed via flow cytometry analysis. n=4. P values were determined using a one-way ANOVA with a Tukey multiple-comparison posttest. H) T cell receptor variable beta chain (TCR Vß) clonotype distribution was measured by flow cytometry of CD8 T cells from mice treated with VSV-Y3 or VSV-Q4. n=4. Mean values have been plotted for clarity. I) Tumors were measured over time and the geometric mean was plotted. n=8. J) Survival curve of VSV-APL-treated mice. n=8 mice per group. Statistical significance was determined via Gehan-Breslow-Wilcoxon test.

RIP-mOVA mice were implanted with B16-OVA-IFNAR^−/−^ tumor cells and then injected with VSV-APL i.t. (Fig. 6A). In this model, inducing an OVA-specific antitumor response, or autoimmunity, results in T cells targeting cells of the pancreas and establishes a diabetic phenotype (Fig. 6B. The most altered, least self APL SIIQFEKL induced the greatest level of autoimmunity consistent with the generation of anti-SIIQFEKL T cells which could also recognize wild type OVA expressed in the pancreas (Fig. 6C). VSV-Q4 treatment generated non-self-specific T cells (anti-Q4) with cross reactivity to SIINFEKL (Fig 6D–E). Flow staining with SIINFEKL and SIIQFEKL tetramers also confirmed the expansion of self-reactive (anti-SIINFEKL) T cells in mice treated with VSV-Y3. These findings suggest that the affinity threshold of Y3 is sufficient for breaking tolerance in this model, but that the Q4 mutation generates heteroclitic epitope-reactive T cells which productively cleared both self and near-self antigen presenting cells. Furthermore, these mono- and dual-reactive T cells differed in expression of exhaustion markers PD1^+^LAG3^+^ and CD39 suggesting that these different subsets underwent different differentiation pathways in the tumor bed possibly contributing to progenitor- and terminal-exhaustion phenotypes (Fig. 6F–G). Further, the T cell populations generated by VSV-Y3 and VSV-Q4 therapy showed variable diversity amongst TCR Vß chains, with the TCRs from VSV-Q4 being predominately Vß5.1/5.2, similar to that of anti-OVA OT-1 TCRs^46^ (Fig. 6H). The presence of these heteroclitic antigen-reactive T cells corresponded with a transient restriction of tumor growth, although this did not equate to significant improvement of therapeutic efficacy (Fig. 6I–J). These data show that the breaking of central tolerance is achievable within our model via generation of T cells reactive against altered self-versions of endogenous TAA which can also react against the unaltered version of the TAA as expressed on the tumor.

Taken together, our findings show that OV monotherapy does not necessarily lead to the development of tumor-specific immunity even when the target TAA is a highly immunogenic, non-tolerized antigen. However, a fundamental understanding of the immunological consequences of OV-driven lympho-editing can inform combinatorial strategies by reaping the benefits of antiviral innate heat while preserving the subdominant antitumor T cell population.

## Discussion

We show here that, although the infiltration of CD8 T cells post-OV therapy is generally considered to be a positive indicator of effective therapy, such infiltrates are likely to represent predominantly antiviral T cells which, at best, may co-exist with antitumor T cells and at worst may actively extinguish pre-existing antitumor T cell responses.

Here, we tested the ability of VSV to convert an immunologically poorly infiltrated cold tumor into a heavily infiltrated hot tumor. We established a model in which the ability of OV therapy to prime and expand an antitumor T cell response was immunologically as good as possible. Thus, in the B16-OVA model we assessed the ability of intratumoral VSV to prime a pre-existing clonal population of naïve antitumor T cells bearing transgenic TCRs for the SIINFEKL epitope of ovalbumin (OT-1) under ideal immunological conditions wherein the tumor antigen (OVA) was a non-self, highly immunogenic protein.

Neither ADU-S100 or Poly I:C, lacking any distracting viral immunogens and both clinically relevant antiviral immune adjuvants triggering the STING/cGAS and TLR3 signaling cascade, respectively, were unable to prime OT-I responses at low-doses despite improved CD8 T cell infiltration into the tumor. Contrary to expectation, VSV-GFP treatment was also insufficient for the priming of OT-1 T cells despite induction of virus-specific T cell expansion, extensive tumor infiltration with activated T cells (cold to hot), and transient restriction of tumor growth. In contrast, a significant population of anti-OVA CD8 T cells was generated *in vivo* when VSV expressed OVA in either the presence, or absence, of OT-1 T cells. These data show that rapid expression of tumor antigens in the presence of an inflammatory viral adjuvant was sufficient for the potent priming of both adoptively transferred and endogenous antitumor T cells and that this antitumor T cell response could co-exist with a potent antiviral T cell response. We also observed that a lower affinity tumor antigen expressed by VSV (VSV-OVA-SIYNFEKL) reduced the expansion of OT-1 T cells while simultaneously increasing the priming of endogenous OVA-reactive T cells compared to VSV-OVA-wt, although we did not see a notable phenotypic difference in the two anti-OVA populations. Taken together, our data here show that even in immunologically favorable conditions, highly inflammatory oncolysis within a cold tumor can generate a hot TME but may be insufficient to prime subdominant antitumor T cells within the context of an immunodominant antiviral response. Moreover, the dominance of the priming of high-affinity antiviral T cells (αVSVN) over the priming of high affinity antitumor T cells (OT-1) following OV therapy in the absence of encoded TAA suggests that levels of TAA present in/released by tumor undergoing oncolysis may be a critical factor in the efficacy of OV therapy as an immunotherapy.

Treating tumors with ADU-S100, an agonist of the STING DNA-sensing pathway^47^, increased the presence of TILs presumably targeting uncharacterized tumor antigens (Fig. 3D) but had little therapeutic effect. CyTOF analysis showed that the phenotype of TILs generated by nonviral immune adjuvant differs significantly from those responding to OV infection. OV-responsive TIL were comprised of fewer terminally exhausted T cells (LAG3^hi^TIM3^hi^) and showed reduced expression of CD39. Most significantly, VSV-OVA therapy rescued a population of progenitor exhausted T cells (LAG3^+^TIM3^−^) which was absent from treatment with VSV alone. These data suggest that this population can co-exist at the nexus of antiviral and antitumor immunity and is responsible, at least in part, for the improvement in therapy associated with VSV oncolysis expressing an associated TAA (Fig. 3G). Therefore, in this model an immunodominant T cell population directly linked to the anti-VSV response is generated, and this dominance can be partially restricted – or potentially shared – when the virus is armed with high-affinity tumor antigen. Studies are underway to determine if this effect is due simply to increased levels of TAA as provided by the VSV-OVA, or to the specific pathways that are induced by viral-mediated TAA presentation.

Of the different OV that we tested, with different levels and quality of virus-induced innate inflammation, only VSV-OVA had the potential to generate both a highly inflammatory environment and potent OVA-specific T cells response which correlated with increased survival. In contrast, inflammation/no TAA (Reovirus) or minimal inflammation/TAA (Ad-OVA) had very small impacts on overall survival (Fig. 4A–E). Aggregated median expression values of CD3 TILs based on markers of activation and exhaustion showed distinct clustering of non-inflammatory conditions (PBS, Ad-GFP, Ad-OVA) and inflammatory viruses, the latter contributing heavily to the depletion of CD4 T cells and endogenous hgp100 antitumor T cells (Fig. 4F). These data confirm the role of innate antiviral mechanisms in both generating potent antiviral T cells responses as well as restricting the priming of antitumor T cells. Interestingly, the generation of antitumor T cells in the presence of only very mild inflammation (Ad-OVA) was insufficient for therapy suggesting that the combination of OV-induced innate immune activation, abundant presence of virus presented TAA, and generation of antitumor T cells responses are necessary for optimal OV immunotherapy.

The demonstration of antagonism between the antiviral and antitumor T cell responses led us to explore combination approaches to selectively expand tumor-specific T cells while preserving the inherent benefits of antiviral inflammation. ICB therapy has become the standard of care for a handful of malignancies such as melanoma, HCC, and small cell lung cancer. However, several remarkable cases of clinical success are overshadowed by the greater rates of transient or non-responding patients^48^. We leveraged the ability of αPD1 ICB to enhance a CD8 T cell response to either viral and/or tumor antigens to study the temporal expansion of these dominant and subdominant T cell populations, respectively. We show that early administration of ICB acted predominantly upon the rapidly expanding antiviral T cells when B16 tumor-bearing mice were co-treated with αPD1 and VSV-OVA; importantly, in this context, OVA was effectively a viral antigen, and this led to a survival outcome of 67% (Fig. 5B). However, using VSV alone or VSV expressing hgp100, no difference between late and early administration of αPD1 was observed. Thus, it seems likely that ICB treatment will preferentially focus on the immunodominant antiviral T cell response and that low affinity TAA are unable to compete for T cell activation in the presence of immunodominant viral antigens even with αPD1 therapy (Fig. 5D–E).

Antigen affinity is at the forefront of T cell therapies aiming to produce a durable tumor-specific T cell response^17^. In the context of viral immunodominance, a high affinity self-antigen (OVA) was sufficient for stimulating tumor-specific T cells, but a lower affinity self-antigen (hgp100) was not. Therefore, we attempted to determine if there is a threshold of TCR affinity for a near self TAA sufficient for breaking central tolerance to both the altered self and unaltered self TAA. Thus, we used the well-characterized OVA antigen in a model system in which OVA is a fully tolerized self-antigen, allowing us to selectively mutate it further from self. RIP-mOVA mice express membrane-bound OVA in pancreatic beta cells and kidney proximal tubular cells, with weak expression in the testes. Pairing this model with VSV-expressed altered peptide ligands of OVA (VSV-APL) bearing differential reactivities to its cognate TCR, we showed that a level of altered self can be reached at which central tolerance is broken to self in the presence of antiviral heat. We used fluorescent tetramers presenting self (SIINFEKL “N4”) and altered-self (SIIQFEKL “Q4”) peptides to identify heteroclitic antigen-reactive TCRs on T cells expanded by VSV-APL therapy. The expression of OVA on pancreatic beta cells allowed us to use the induction of diabetic autoimmunity as our readout for successful breaking of tolerance. In this respect, the greatest induction of autoimmunity was generated from the least-self variant Q4 (Fig. 6C). Strikingly, we observed an expansion of anti-self (anti-N4) T cells following treatment with VSV-Y3 and at much lower levels with VSV-Q4. These data suggest that the breaking of tolerance to a self TAA is possible by epitope engineering in which a relatively narrow deviation from self is achieved and is associated with exceeding an affinity threshold of the near self TAA for T cells; however, if the antigen is too far from self the ability of generating T cells reactive to self-antigens is nullified. A small subpopulation of T cells specifically targeting the Q4 ligand were also found to be cross-reactive to endogenous N4 antigen (Fig 6D–E), and the notable difference in clonal populations based on TAA affinity suggests that TCRs from cross-reactive and N4 reactive T cells are physiologically distinct (Fig. 6H). Our data show that this Q4 and heteroclitic antigen-reactive T cells restricted tumor growth (Fig 6I). These data open a novel strategy by which OV therapy can be used to break tolerance to self TAA by priming a population of heteroclitic near-self-reactive T cells which can escape negative selection in the thymus but which also react against self TAA expressed by the tumor. Moreover, we show that these heteroclitic antitumor T cells could co-exist with immunodominant antiviral T cells leading to an impact on tumor growth. We propose that by encoding multiple heteroclitic, near self TAA within OV^25,49^, it will be possible to optimize the generation of antitumor T cell responses even in the presence of highly immune-distracting antiviral T cell responses.

In summary, our data here lays the foundation for a paradigm shift in the field of OV mono- and combination therapies which addresses the role of immunodominance as it pertains to tumor-specific immunity. Here we show that 1) the widely proposed mechanisms by which antitumor T cell responses are proposed to be generated as a result of highly inflammatory oncolysis should not be taken for granted 2) that the antiviral T cell response may occlude an antitumor response, even in the presence of immunogenic TAA and 3) that it will be possible to overcome the antiviral attrition of antitumoral T cells by engineering deviation from self into viral expressed TAA to generate heteroclitic antitumor T cells. These findings elucidate strategies for overcoming the negative effect of viral dominance while still preserving the vector’s valuable adjuvant properties in cold tumors.

## Online Methods

### Cell lines and viruses

B16 murine melanoma cells and MC38 murine colon adenocarcinoma cells were originally obtained from ATCC and maintained in complete Dulbecco’s Modified Eagle Medium (10% Fetal Bovine Serum, 1% penicillin-streptomycin). B16-ova cells were derived from B16 cells stably transduced by cDNA encoding ovalbumin and neomycin resistance and positive clones were selected 5μg/ml Neomycin (G148, Corning 61–234-RG)^50^. MC38-ova were generated via lentiviral transduction using pHR-SIIN-ovalbumin-puromycin vector and maintained in media containing 1.25ug/ml puromycin (Sigma P9620). B16-ova-IFNAR^−/−^ cells were generated by Cas9 RNP mediated editing. Two IFNAR1 targeting crRNAs (Mm.Cas9.IFNAR1.1.AA (TCAGTTACACCATACGAATC) and Mm.Cas9. IFNAR1.1.AB (GCTTCTAAACGTACTTCTGG)) and Alt-R CRISPR-Cas9 Negative Control crRNAs #1 and #2 were ordered from Integrated DNA Technologies (IDT). Knockout protocol was performed as previously reported.^26^

VSV expressing ovalbumin or green fluorescent protein (VSV-GFP) were rescued from the pXN2 cDNA plasmid using a previously established reverse genetics system and propagated in BHK cells^20,51^. Recombinant ovalbumin plasmid DNA was synthesized from ovalbumin DNA using A2 (For: AGGCCTTGAGCAGCTTGAGAGTgcAATCAACTTTGA, Rev: GTCCATTCAGTCAGTTTTTCAAAGTTGATTgcACTCTCAAGC), Y3 (For: AGGCCTTGAGCAGCTTGAGAGTATAtaCAACTTTGAAAAAC, Rev: TGGTCCATTCAGTCAGTTTTTCAAAGTTGtaTATACTCTCAAG), and Q4 (For: AGGCCTTGAGCAGCTTGAGAGTATAATCcaaTTTGAAAAAC, Rev: ACTGGTCCATTCAGTCAGTTTTTCAAAttgGATTATACTCTCAAG) primers. Restriction double digest was performed using Nhe1 and XhoI restriction sites on ova and the insert was ligated into the VSV-XN_2 vector. (For: TAAGCACTCGAGATGGGCTCCATCG, Rev: ACTGGTCCATTCAGTCAGTTTTTCAAAttgGATTATACTCTCAAG)

BHK cells were infected with VSV at an MOI 0.01 for 24 h and supernatant was collected and passed through a 0.22μm filter to remove debris. Supernatant was then purified through a 10% sucrose cushion 2x via ultracentrifugation at 27000rpm (Beckman Coulter Optima XPN-100) for 1hr. Monoclonal vectors were rescued via noble agar plaque purification (2x) as previously described^52^. Virus stock titers were determined by standard plaque assay of serially diluted supernatants on BHK cells. Reovirus type 3 (Dearing strain) was obtained from Oncolytics Biotech (Calgary, AB, Canada) and stock was used directly for in vivo studies without propagation. Replication defective Adenovirus serotype 5 expressing ovalbumin was kindly gifted from Michael Barry (Mayo Clinic).

### In vitro infection and multi-step growth curve

BHK cells were plated at 1e5 cells in a 24 well plate in complete Dulbecco’s Modified Eagle Medium (10% Fetal Bovine Serum, 1% penicillin-streptomycin) and incubated at 37C until confluent. Monolayer was infected with VSV at MOI 0.01 in Serum-Free DMEM for 1 hr. Infection media was removed and replaced with complete DMEM and supernatants were collected at respective timepoints, clarified, and frozen until titration on BHK cells as before.

### Mice

Female C57Bl/6J (Strain #:**000664**), B6.SJL-Ptprca Pepcb/BoyJ (Strain #:**002014**), and C57BL/6-Tg(Ins2-TFRC/OVA)296Wehi/WehiJRIP-mOVA (Strain #:**005431**) were obtained from The Jackson Laboratory (Bar Harbor, ME). Mice were 6–8 weeks of age upon receipt and maintained in a specific pathogen-free BSL2 biohazard facility. C57BL/6-Tg(TcraTcrb)1100Mjb/J OT1 mice (Strain #:**003831**) were originally obtained from the Jackson Laboratory and were bred at the Mayo Clinic. Splenocytes and lymph nodes from female mice were harvested between 8 and 12 weeks of age for adoptive transfer experiments. All animal studies were conducted in accordance with and approved by the Institutional Animal Care and Use Committee at Mayo Clinic.

### OT-1 T cell preparation and administration

Spleens and lymph nodes were harvested from OT-1 mice and purified using the Miltenyi Biotec CD8a T cell isolation kit (CAS 130–104-075) as per the protocol and washed 2x in PBS immediately prior to i.v. injection by tail vein for *in vivo* studies.

### In vivo studies

C57Bl/6J, PepboyJ, and RIP-mOVA mice were challenged subcutaneously with 5e5 B16-ova, B16-ova-IFNAR^−/−^, or MC38-ova tumor cells in 100μL PBS (Corning). Once tumors reached approx. 0.2cm in diameter, treatments were administered i.t. For immune adjuvant studies, tumors were treated with an immunogenic dose of 10ug ADU-S100 or 50ug Poly I:C. VSV and Adenoviral vectors were administered up to 3x with 5e8pfu in 50ul PBS per dose i.t., and Reovirus was administered up to 3x at 4.5e8 pfu in 50ul per dose i.t. Mice received up to 3 doses of 100ug aPD1 checkpoint inhibitor (Clone 29F.1A12 BioXcell BE0273) in 100ul PBS intraperitoneally (i.p.) per treatment. Tumor volume was calculated using the following equation: (LengthxWidth^2^)/2

### Flow cytometry

Single cell suspensions of spleens, lymph nodes, blood, and tumors were generated from euthanized mice and immediately processed for flow cytometry studies. Tumors were weighed and digested with DNAse I (Sigma) and Liberase TL (Roche) for 30 min in a 37°C water bath. Spleens, tumors, and blood were subjected to ACK red blood cell lysis buffer. Cells were stained for surface markers, washed, and fixed in 4% formaldehyde as previously described^53^. Samples were analyzed in the Mayo Clinic Flow Cytometry Core (Rochester, MN) using a Cytek Aurora spectral flow cytometer with SpectroFlo (V3.1.0) software for unmixing and Flowjo (V10.1) for data analysis. Information for flow cytometry reagents and working concentrations can be found in Online Materials Table 1.

For intracellular staining and detection of IFNy, cell suspensions were first cultured in RPMI + IL2 (1:1000) at 37°C + 5% CO2 for no more than 12 hours. Suspensions were then stimulated with viral or tumor peptides and Golgi plug 1:1000 (BD) as denoted at a peptide concentration of 1ug/ml for 4 hours. Cells were then stained and fixed using BD Cytofix/Cytoperm plus with BD Golgi Plug kit (BD Biosciences 555028). Peptides were obtained from the Mayo Clinic Proteomics Core Facility (Rochester, MN).

TCR Vß analysis was performed using the Anti-Mouse TCR Vß Screening Panel (BD Pharmingen #557004) at a 1:5 dilution.

Tetramers for Ova_257–264_ (SIINFEKL)-APC, hgp100_25–33_(KVPRNQDWL)-BB515, VSVN_52–59_ (RGYVYQGL)-BV421 and Reo μ1_133–140_ (VSPKYSDL)-PE were obtained from the NIH Tetramer Core Facility. Tetramers were used at a concentration of 1:100 and incubated at 4°C for 30 min.

### Statistical analyses

Statistical analyses were performed using GraphPad Prism 10.3.1 software. One-way or two-way ANOVAs with a Tukey’s post-hoc multiple comparisons tests were used where applicable. Survival data were assessed using Gehan-Breslow-Wilcoxon test and P-values have been set at p=<0.05 and ns=>0.05. Error bars denote the SEM of samples. Figures were generated using GraphPad Prism 10.3.1 software and Biorender. Cluster plot analysis was performed using R CATALYST package in R.4.3.1 described by Nowicka et. al.^54^

Sample sizes were determined and power analysis performed based on those reported in previous publications^20,26^. Normal data distribution was assumed but not officially tested. Mice were randomized to treatment groups after tumor implantation. Pre-established exclusion criteria from mouse survival studies included removal of animals with failed tumor engraftment and those which were euthanized due to endpoint criteria outside of tumor sizes reaching 1cm in diameter. Data from spleen samples with poor overall viability from Fig. 3B, 5C, and 5E were excluded from analysis.

## References

[1] Kaufman H. L., Kohlhapp F. J. & Zloza A. Oncolytic viruses: a new class of immunotherapy drugs. Nat Rev Drug Discov 14, 642–662 (2015). 10.1038/nrd4663 26323545 PMC7097180

[2] Russell L. & Peng K. W. The emerging role of oncolytic virus therapy against cancer. Chin Clin Oncol 7, 16 (2018). 10.21037/cco.2018.04.04 29764161 PMC6557159

[3] Shalhout S. Z., Miller D. M., Emerick K. S. & Kaufman H. L. Therapy with oncolytic viruses: progress and challenges. Nat Rev Clin Oncol 20, 160–177 (2023). 10.1038/s41571-022-00719-w 36631681

[4] Russell S. J., Bell J. C., Engeland C. E. & McFadden G. Advances in oncolytic virotherapy. Commun Med-London 2 (2022). 10.1038/s43856-022-00098-4 PMC9053215 35603308

[5] Malogolovkin A. Combinatorial Approaches for Cancer Treatment Using Oncolytic Viruses: Projecting the Perspectives through Clinical Trials Outcomes. Viruses 13 (2021). 10.3390/v13071271 PMC8309967 34209981

[6] Mehrabadi A. Z. Overview of the pre-clinical and clinical studies about the use of CAR-T cell therapy of cancer combined with oncolytic viruses. World J Surg Oncol 20 (2022). 10.1186/s12957-021-02486-x PMC8756705 35027068

[7] Shaw A. R. & Suzuki M. Oncolytic Viruses Partner With T-Cell Therapy for Solid Tumor Treatment. Frontiers in Immunology 9 (2018). 10.3389/fimmu.2018.02103 PMC6160535 30298067

[8] Santos Apolonio J. Oncolytic virus therapy in cancer: A current review. World J Virol 10, 229–255 (2021). 10.5501/wjv.v10.i5.229 34631474 PMC8474975

[9] Chen L., Zuo M., Zhou Q. & Wang Y. Oncolytic virotherapy in cancer treatment: challenges and optimization prospects. Frontiers in Immunology 14, undefined-undefined (2023). 10.3389/fimmu.2023.1308890 PMC10758479 38169820

[10] Kim S. K. & Cho S. W. The Evasion Mechanisms of Cancer Immunity and Drug Intervention in the Tumor Microenvironment. Front Pharmacol 13, 868695 (2022). 10.3389/fphar.2022.868695 35685630 PMC9171538

[11] Motz G. T. & Coukos G. Deciphering and reversing tumor immune suppression. Immunity 39, 61–73 (2013). 10.1016/j.immuni.2013.07.005 23890064 PMC3782392

[12] Stone J., Chervin A. & Kranz D. T-cell receptor binding affinities and kinetics: impact on T-cell activity and specificity. Immunology 126, 165–176 (2009). 10.1111/j.1365-2567.2008.03015.x 19125887 PMC2632691

[13] Zhong S. T-cell receptor affinity and avidity defines antitumor response and autoimmunity in T-cell immunotherapy. Proceedings of the National Academy of Sciences (PNAS) 110, 6973–6978 (2013). 10.1073/pnas.1221609110 PMC3637771 23576742

[14] Chen W. & McCluskey J. Immunodominance and immunodomination: critical factors in developing effective CD8+ T-cell-based cancer vaccines. Adv Cancer Res 95, 203–247 (2006). 10.1016/S0065-230X(06)95006-4 16860659

[15] Gong N. Enhancing in situ cancer vaccines using delivery technologies. Nature Reviews Drug Discovery 23, 607–625 (2024). 10.1038/s41573-024-00974-9 38951662

[16] McNally J. Attrition of Bystander CD8 T Cells during Virus-Induced T-Cell and Interferon Responses. Journal of Virology 75, 5965–5976 (2001). 10.1128/JVI.75.13.5965-5976.2001 11390598 PMC114312

[17] Miller A., Bahmanof M., Zehn D., Cohen E. W. & Schoenberger S. Leveraging TCR Affinity in Adoptive Immunotherapy against Shared Tumor/Self-Antigens. Cancer Immunology Research 7, 40–49 (2019). 10.1158/2326-6066.CIR-18-0371 30482746 PMC7793606

[18] Badr M., Zhang Z., Tai X. & Singer A. CD8 T cell tolerance results from eviction of immature autoreactive cells from the thymus. Science (AAAS) 382, 534–541 (2023). 10.1126/science.adh4124 PMC11302524 37917689

[19] ElTanbouly M. & Noelle R. Rethinking peripheral T cell tolerance: checkpoints across a T cell’s journey. Nature Reviews Immunology 21, 257–267 (2020). 10.1038/s41577-020-00454-2 PMC12536352 33077935

[20] Diaz R. M. Oncolytic immunovirotherapy for melanoma using vesicular stomatitis virus. Cancer Res 67, 2840–2848 (2007). 10.1158/0008-5472.CAN-06-3974 17363607

[21] Rommelfanger D. M. Systemic combination virotherapy for melanoma with tumor antigen-expressing vesicular stomatitis virus and adoptive T-cell transfer. Cancer Res 72, 4753–4764 (2012). 10.1158/0008-5472.CAN-12-0600 22836753 PMC3893932

[22] Driscoll C. B. APOBEC3B-mediated corruption of the tumor cell immunopeptidome induces heteroclitic neoepitopes for cancer immunotherapy. Nat Commun 11, 790 (2020). 10.1038/s41467-020-14568-7 32034147 PMC7005822

[23] Huff A. L. Vesicular Stomatitis Virus Encoding a Destabilized Tumor Antigen Improves Activation of Anti-tumor T Cell Responses. Mol Ther 28, 2540–2552 (2020). 10.1016/j.ymthe.2020.08.013 32877695 PMC7705043

[24] Overwijk W. W. Tumor regression and autoimmunity after reversal of a functionally tolerant state of self-reactive CD8+ T cells. J Exp Med 198, 569–580 (2003). 10.1084/jem.20030590 12925674 PMC2194177

[25] Webb M. J. Expression of tumor antigens within an oncolytic virus enhances the anti-tumor T cell response. Nat Commun 15, 5442 (2024). 10.1038/s41467-024-49286-x 38937436 PMC11211353

[26] Evgin L. Oncolytic virus-derived type I interferon restricts CAR T cell therapy. Nat Commun 11, 3187 (2020). 10.1038/s41467-020-17011-z 32581235 PMC7314766

[27] Shim K. Inhibitory Receptors Induced by VSV Viroimmunotherapy Are Not Necessarily Targets for Improving Treatment Efficacy. Molecular Therapy 25, 962–975 (2017). 10.1016/j.ymthe.2017.01.023 28237836 PMC5383647

[28] Jiang H. Oncolytic Adenovirus and Tumor-Targeting Immune Modulatory Therapy Improve Autologous Cancer Vaccination. Cancer Research 77, 3894–3907 (2017). 10.1158/0008-5472.CAN-17-0468 28566332 PMC5549681

[29] Puigdelloses M. CD137 and PD-L1 targeting with immunovirotherapy induces a potent and durable antitumor immune response in glioblastoma models. Journal for ImmunoTherapy of Cancer 9, e002644-undefined (2021). 10.1136/jitc-2021-002644 34281988 PMC8291319

[30] Jeon Y. H. Oncolytic Vaccinia Virus Augments T Cell Factor 1-Positive Stem-like CD8(+) T Cells, Which Underlies the Efficacy of Anti-PD-1 Combination Immunotherapy. Biomedicines 10 (2022). 10.3390/biomedicines10040805 PMC9027961 35453555

[31] Burman B., Pesci G. & Zamarin D. Newcastle Disease Virus at the Forefront of Cancer Immunotherapy. Cancers 12, 3552-undefined (2020). 10.3390/cancers12123552 33260685 PMC7761210

[32] Zamarin D. Enhancement of Oncolytic Properties of Recombinant Newcastle Disease Virus Through Antagonism of Cellular Innate Immune Responses. Molecular Therapy 17, 697–706 (2009). 10.1038/mt.2008.286 19209145 PMC2835121

[33] Jeon Y. H. Oncolytic Vaccinia Virus Augments T Cell Factor 1-Positive Stem-like CD8 T Cells, Which Underlies the Efficacy of Anti-PD-1 Combination Immunotherapy. Biomedicines 10 (2022). 10.3390/biomedicines10040805 PMC9027961 35453555

[34] Xie D. Oncolytic adenoviruses expressing checkpoint inhibitors for cancer therapy. Signal Transduct Target Ther 8, 436 (2023). 10.1038/s41392-023-01683-2 38016957 PMC10684539

[35] Sivick K. Magnitude of Therapeutic STING Activation Determines CD8+ T Cell-Mediated Anti-tumor Immunity. Cell Reports 25, 3074–3085.e3075 (2018). 10.1016/j.celrep.2018.11.047 30540940

[36] Aznar M. A. Immunotherapeutic effects of intratumoral nanoplexed poly I:C. Journal for ImmunoTherapy of Cancer 7, 116-undefined (2019). 10.1186/s40425-019-0568-2 31046839 PMC6498680

[37] Lau J. Tumour and host cell PD-L1 is required to mediate suppression of anti-tumour immunity in mice. Nature Communications 8, 14572-undefined (2017). 10.1038/ncomms14572 PMC5321797 28220772

[38] Moesta A., Li X.-Y. & Smyth M. Targeting CD39 in cancer. Nature Reviews Immunology 20, 739–755 (2020). 10.1038/s41577-020-0376-4 32728220

[39] Zehn D., Lee S. & Bevan M. Complete but curtailed T-cell response to very low-affinity antigen. Nature 458, 211–214 (2009). 10.1038/nature07657 19182777 PMC2735344

[40] Vinay D. & Kwon B. CD11c+CD8+ T cells: Two-faced adaptive immune regulators. Cellular Immunology 264, 18–22 (2010). 10.1016/j.cellimm.2010.05.010 20620256

[41] Fiorentini S. CD11b Expression Identifies CD8+CD28+ T Lymphocytes with Phenotype and Function of Both Naive/Memory and Effector Cells. Journal of Immunology 166, 900–907 (2001). 10.4049/jimmunol.166.2.900 11145666

[42] Berkeley R. Antibody-Neutralized Reovirus Is Effective in Oncolytic Virotherapy. Cancer Immunology Research 6, 1161–1173 (2018). 10.1158/2326-6066.CIR-18-0309 30209061

[43] Rajani K. Combination Therapy With Reovirus and Anti-PD-1 Blockade Controls Tumor Growth Through Innate and Adaptive Immune Responses. Molecular Therapy 24, 166–174 (2016). 10.1038/mt.2015.156 26310630 PMC4754544

[44] Schuelke M. Phase I trial of sargramostim/pelareorep therapy in pediatric patients with recurrent or refractory high-grade brain tumors. Neuro-Oncology Advances 4, undefined-undefined (2022). 10.1093/noajnl/vdac085 PMC9268737 35821679

[45] Holzgruber J. Type I interferon signaling induces melanoma cell-intrinsic PD-1 and its inhibition antagonizes immune checkpoint blockade. Nature Communications 15, 7165-undefined (2024). 10.1038/s41467-024-51496-2 PMC11347607 39187481

[46] Rubinstein M. Transfer of TCR Genes into Mature T Cells Is Accompanied by the Maintenance of Parental T Cell Avidity. Journal of Immunology 170, 1209–1217 (2003). 10.4049/jimmunol.170.3.1209 12538678

[47] Barber G. STING: infection, inflammation and cancer. Nature Reviews Immunology 15, 760–770 (2015). 10.1038/nri3921 PMC5004891 26603901

[48] Sun Q. Immune checkpoint therapy for solid tumours: clinical dilemmas and future trends. Signal Transduction and Targeted Therapy 8, 320-undefined (2023). 10.1038/s41392-023-01522-4 37635168 PMC10460796

[49] Pulido J. Using virally expressed melanoma cDNA libraries to identify tumor-associated antigens that cure melanoma. Nature Biotechnology 30, 337–343 (2012). 10.1038/nbt.2157 PMC3891505 22426030

[50] Linardakis E. Enhancing the efficacy of a weak allogeneic melanoma vaccine by viral fusogenic membrane glycoprotein-mediated tumor cell-tumor cell fusion. Cancer Res 62, 5495–5504 (2002). 12359759

[51] Obuchi M., Fernandez M. & Barber G. Development of Recombinant Vesicular Stomatitis Viruses That Exploit Defects in Host Defense To Augment Specific Oncolytic Activity. Journal of Virology 77, 8843–8856 (2003). 10.1128/JVI.77.16.8843-8856.2003 12885903 PMC167243

[52] Lun X. Effects of Intravenously Administered Recombinant Vesicular Stomatitis Virus (VSV ΔM51 ) on Multifocal and Invasive Gliomas. JNCI Journal of the National Cancer Institute 98, 1546–1557 (2006). 10.1093/jnci/djj413 17077357

[53] Diaz R. Oncolytic Immunovirotherapy for Melanoma Using Vesicular Stomatitis Virus. Cancer Research 67, 2840–2848 (2007). 10.1158/0008-5472.CAN-06-3974 17363607

[54] Nowicka M. CyTOF workflow: differential discovery in high-throughput high-dimensional cytometry datasets. F1000Research 6, 748-undefined (2019). 10.12688/f1000research.11622.3 PMC5473464 28663787

